# Rodzice Uchylający Się Od Szczepienia Ochronnego Dziecka w Okresie Noworodkowym ‒ Analiza Postaw^*^

**DOI:** 10.34763/devperiodmed.20182204.315322

**Published:** 2019-01-14

**Authors:** Maria Wilińska, Małgorzata Warakomska

**Affiliations:** 1 Klinika Neonatologii, Centrum Medyczne Kształcenia Podyplomowego, Warszawa, Polska

**Keywords:** noworodek, szczepienie ochronne, odmowa szczepienia, newborn, vaccinations, avoidance of vaccination

## Abstract

*Uchylanie się rodziców od szczepień ochronnych jest zjawiskiem narastającym i wiedzie do pogorszenia sytuacji epidemiologicznej w zakresie chorób objętych profilaktyką czynną*.

*Celem badania była analiza postaw rodziców uchylających się od szczepień ochronnych u noworodków w oddziale neonatologicznym*.

**Materiał i metody:**

*Badanie ankietowe analizujące postawy rodziców uchylających się od szczepienia dziecka po jego urodzeniu się przeprowadzono w ośrodku III poziomu referencyjnego. Gromadzono dane demograficzne rodziców, powody ich decyzji, źródła informacji oraz stan realizacji szczepień ochronnych dziecka po ukończeniu przez nie 6 miesiąca życia*.

**Wyniki:**

*Stwierdzono narastającą w latach 2015-2017 liczbę rodziców uchylających się od szczepień (odpowiednio 1,58%, 2,54%, 2,83%). Byli to rodzice dojrzali wiekowo (31,5-34,5 lat), w większości z wyższym wykształceniem (93%). 63% posiadała więcej niż jedno dziecko. Wśród wielodzietnych 67% rodziców szczepiło poprzednie dzieci. Jako powód wstrzymania się od szczepienia podawano brak rzetelnej informacji medycznej od personelu, negatywne opinie z internetu i od innych rodziców*.

**Wnioski:**

*Niewystarczająca aktywność personelu medycznego i silny wpływ łatwo dostępnych w mediach społecznościowych opinii antyszczepionkowych są przyczyną nieoptymalnej realizacji programu szczepień ochronnych. Konieczne jest propagowanie rzetelnej wiedzy na temat zagrożeń epidemiologicznych chorób objętych szczepieniami ochronnymi oraz umiejętnego jej przekazywania z wykorzystaniem wszelkich dostępnych dróg przekazu*.

## Abstract (en)

*Parents’ avoidance of vaccination is a growing phenomenon and leads to the deterioration of the epidemiological situation regarding diseases included in active prevention programs*.

*The aim of the study was to analyze the attitudes of parents who avoid vaccination in newborns*.

**Material and methods:**

*The study included parents who refused to perform vaccination in their newborn. A survey analyzing the attitudes of parents avoiding vaccination in newborns was performed in a tertiary referral hospital in the years 2015-2017. We gathered information concerning their demographic data, comprising the reasons for their decision, information sources and the implementation of vaccination in a child after six months*.

**Results:**

*We observed an increase in the number of parents avoiding vaccination in the years 2015-2017 (1.58%, 2.54%, 2.83% respectively). The parents were mature (age 31.5-34.5 years), usually with university education (93%). 63% had more than one child. In large families, 67% of the parents had vaccinated their older children. The lack of honest medical information from the personnel, negative opinions from the Internet and other parents were the reasons for avoiding vaccination*.

**Conclusions:**

*The insufficient activity of the medical personnel and the strong influence of anti-vaxxers’ opinions, which is easily accessible on social media, are the reasons for the nonoptimal implementation of the vaccination program. It is necessary to spread honest knowledge about the epidemiological threats concerning vaccine-preventable diseases and develop a skillful way of distributing it through all the possible ways of communication*.

## Wstęp

Poprawa przeżywalności dzieci na świecie jest w olbrzymim stopniu związana z wprowadzaniem immunizacji czynnej już od okresu noworodkowego. Skuteczność szczepień ochronnych w eradykacji lub zminimalizowaniu zachorowalności została udokumentowana w raportach Światowej Organizacji Zdrowia (ang. *World Health Organization − WHO*) i Funduszu Narodów Zjednoczonych na rzecz Dzieci (*ang. United Nations International Children’s Emergency Fund − UNICEF*). Eksperci WHO uważają, że dalsza poprawa w zakresie zasięgu szczepień pozwoliłaby uniknąć około 1,5 miliona zgonów na świecie. Jednakże, pomimo zorganizowanego systemu immunizacji czynnej, aktualnie ponad 19 milionów dzieci na świecie nie otrzymuje podstawowych szczepionek ochronnych [1, 2].

Równolegle, na całym świecie obserwuje się zjawisko narastania liczby rodziców uchylających się od szczepień ochronnych [3, 4, 5].

Z danych Państwowego Zakładu Higieny wynika, że w roku 2016 w Polsce od szczepień ochronnych uchyliło się 23 147 osób. W zależności od województwa, jest to wskaźnik 0,10 do 0,65 na 1000 osób do 19. roku życia. W stosunku do roku 2010 jest to wzrost niemal 8-krotny [6].

Rodzicom brakuje rzetelnej i prawidłowo podanej informacji na temat szczepień ochronnych [7]. Szczepienia ochronne dziecka powinny być przedmiotem aktywnej edukacji rodziców już podczas ciąży. Nie jest jasne, jak w dalszej opiece nad dzieckiem jest realizowana wstępna deklaracja niektórych rodziców o zaledwie odsunięciu w czasie szczepienia ochronnego ich dziecka.

Dobre zrozumienie zarówno przyczyn, jak i kontekstu decyzji rodziców o uchylaniu się od szczepień ochronnych w okresie noworodkowym jest ważnym krokiem do opracowania skutecznych strategii poprawy realizacji programu szczepień ochronnych.

Celem badania było

1. scharakteryzowanie rodziców odmawiających szczepień ochronnych noworodka
2. analiza realizacji programu immunizacji czynnej w okresie do ukończenia 6 miesiąca życia u tych niemowląt.

## Materiał

Badanie przeprowadzono w Klinice Neonatologii Centrum Medycznego Kształcenia Podyplomowego (CMKP) w Warszawie w okresie 01.01.2015-31.12.2017. Badaniem objęto wszystkie kolejno rodzące się noworodki i ich rodziców. Dalsza szczegółowa analiza dotyczyła rodziców, którzy odmówili wykonania szczepienia ochronnego.

## Metoda

Źródłem danych była dokumentacja medyczna pacjentów oraz rutynowo sporządzana lista noworodków nieszczepionych. Rodzice każdorazowo otrzymywali informację o celu szczepienia, skuteczności i bezpieczeństwie zabiegu oraz ryzyku nieszczepienia dla dziecka, rodziny i społeczeństwa. Kolejny kontakt z rodzicami nastąpił po upływie co najmniej 6 miesięcy od urodzenia dziecka. Informacje od rodziców na temat ich postaw i realizacji szczepień u dziecka pozyskiwano telefonicznie, na podstawie odrębnie stworzonej listy pytań w ankiecie.

Analizie poddano rodzaj decyzji (odroczenie, odmowa szczepienia ochronnego), wiek rodziców, ich wykształcenie, zatrudnienie i historię szczepień posiadanych dzieci. Pytano o źródła wiedzy, rodzaj i motywację podejmowanych decyzji. W każdej rozmowie rodzicom przedstawiano cel badania oraz pozyskano zgodę na wykorzystanie informacji dla realizacji celu badania.

Na realizację badania pozyskano zgodę Komisji Bioetycznej przy CMKP Nr 50/PB/2016.

## Wyniki

W latach 2015, 2016 i 2017 urodziło się w Klinice Neonatologii kolejno 1961, 1851 oraz 1913 noworodków. Odsetek niezaszczepionych wynosił odpowiednio 1,58%, 2,54% oraz 2,83% noworodków. Odmowa wykonania szczepienia ochronnego dotyczyła wyłącznie noworodków donoszonych (ryc. 1).

Spośród 132 rodziców, którzy odmówili szczepienia dziecka, kontakt telefoniczny uzyskano ze 125 osobami. Pięcioro rodziców odmówiło udzielenia odpowiedzi. Materiał do dalszej analizy stanowiły dane pozyskane od 120 rodziców.

Obserwowano dwie postawy rodziców uchylających się od szczepień ochronnych noworodka: odmowę lub odroczenie. Mediana wieku matki odmawiającej zaszczepienia dziecka wynosiła 31,5 lat, ojca 32 lata. Rodzice odraczający szczepienie byli nieco starsi. Większość z nich posiadało więcej niż jedno dziecko w rodzinie. Znakomita większość rodziców cechowała się wyższym wykształceniem. Niemal wszyscy byli aktywni zawodowo. Trzy czwarte rodziców, dla których obecne dziecko było kolejnym w rodzinie, szczepiło poprzednie dzieci (tab. I).

**Ryc. 1:**
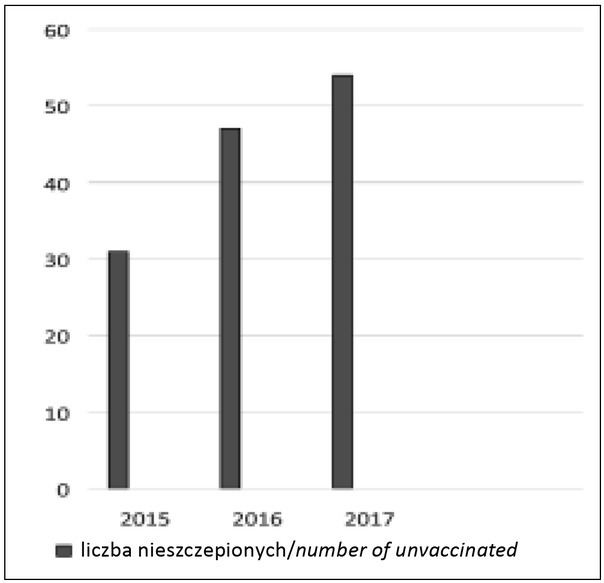
Liczba nieszczepionych noworodków w latach 2015-2017 w Klinice Neonatologii CMKP. *Fig. 1. The number of unvaccinated newborns in the Neonatology Department Centre of Medical Postgraduate Education in 2015-2017*.

Połowa rodziców uchylających się od szczepienia dziecka w oddziale noworodkowym czerpała informacje z internetu oraz od lekarza rodzinnego. Co trzeci rodzic czytał informacje organizacji antyszczepionkowych. Tylko co piąty czerpał wiedzę z fachowej literatury medycznej. Co dziesiąty rodzic rozmawiał na ten temat z rodziną czy znajomymi.

Większość rodziców deklarowała w oddziale noworodkowym odroczenie szczepienia dziecka, tylko 20% zdecydowanie odmówiła zaszczepienia dziecka w ogóle i dotyczyło to w większości obydwu szczepień, BCG i p/WZW B (tab. II).

## Przesłanki podjęcia decyzji o nieszczepieniu dziecka w oddziale noworodkowym

W grupie odraczającej szczepienia ochronne rodzice podkreślali przekonanie o niesprecyzowanym większym bezpieczeństwie nieszczepienia dziecka (54%). 15% rodziców nie widziało większego zagrożenia zachorowaniem na chorobę objętą szczepieniem ochronnym. Co piąty rodzic przyjął taką radę z otoczenia, nie wnikając w szczegółowe powody. Co czwarty rodzic podkreślał ogólną niedojrzałość immunologiczną dziecka i związaną z tym słabą lub nieprzewidywalną reakcję na szczepienie.

Rodzice zdecydowanie odmawiający szczepienia podkreślali przekonanie o ryzyku na zdrowie dziecka związanym z podaniem preparatu. Podawali niekorzystne doświadczenia w rodzinie kojarzone z podaniem szczepionki. Wielu rodziców twierdziło, że nie uzyskało od personelu medycznego wyczerpującej informacji o skuteczności i bezpieczeństwie szczepienia. Inni podkreślali interes komercyjny firm produkujących szczepionki, a nawet interes finansowy personelu medycznego związany z realizacją programu szczepień ochronnych (tab. III).

**Table I:** Characteristics of parents evading newborn immunization. Tabela I. Charakterystyka rodziców uchylających się od szczepienia noworodka.

Charakterystyka grupy *Characteristics of the group*	Wartość *Value*
Wiek rodziców uchylających się od szczepienia (lata) *Age of parents evading vaccinations (years)*	
Matka odraczająca	32
*Mother avoiding*	
Matka odmawiająca	31,5
*Mother refusing*	
Ojciec odraczający	34,5
*Father avoiding*	
Ojciec odmawiający	32
*Father refusing*	

Liczba dzieci w rodzinie (% grupy) *Number of children in the family (% of group)*	
jedno	44 (37)
*one*	
dwoje	52 (43)
*two*	
troje i więcej	24 (20)
*three and more*	

Wykształcenie *Education*	
podstawowe	0 (0)
*basic*	
średnie	8 (7)
*average*	
wyższe	112 (93)
*higher*	
Zatrudnienie (% grupy) *Employment (% of group)*	
oboje pracujący	108 (90)
*both working*	
jeden rodzic pracujący	12 (10)
*one parent working*	

Szczepienie poprzednich dzieci (% grupy)* Previous children vaccinated (% of group)*	
tak	51 (67)
*yes*	
nie	20 (26)
*no*	
częściowo	5 (7)
*partly*	

Główne źródła wiedzy (możliwość wielokrotnej odpowiedzi)	
*Main sources of knowledge (multiple answer option)*	
internet	60 (50)
*internet*	
lekarz rodzinny	56 (47)
*family doctor*	
rodzina, znajomi	12 (10)
*family, friends*	
organizacje antyszczepionkowe	40 (33)
*anti-vaccine organizations*	
literatura medyczna	28 (23)
*medical literature*	

*nie dotyczy – 44 *not applicable – 44

**Table II:** The type of decision regarding vaccination. Tabela II. Rodzaj podejmowanej decyzji co do szczepienia ochronnego.

Treść decyzji (% grupy) *The content of the decision (% of the group)*	Liczność grupy (%) *Group size (%)*
odroczenie	96 (80)
*postponement*	
odmowa	24 (20)
*refusal*	
dotyczy tylko BCG	4 (3,33)
*applies only to BCG*	
dotyczy tylko p/WZW B	4 (3,33)
*applies only to anti-Hepatitis B*	
dotyczy obu szczepień	16 (13,33)
*applies to both vaccinations*	

## Szczepienia ochronne w pierwszym półroczu życia dziecka ‒ analiza wykonalności

Analiza wykonania szczepień ochronnych w pierwszym półroczu życia dziecka w grupie rodziców uchylających się od szczepienia w okresie noworodkowym wykazała zgodność z decyzją podjętą w okresie noworodkowym. Co piąta rodzina z tej grupy trwała w decyzji wstrzymania się od wszystkich szczepień ochronnych. 80% rodziców zrealizowało szczepienia ochronne u dziecka z odroczeniem (tab. IV).

Stopień opóźnienia tej decyzji był bardzo zróżnicowany (dane nie prezentowane).

Większość rodziców trwała w przekonaniu o słuszności decyzji podjętej w okresie noworodkowym dziecka. Ośmioro rodziców poczuło się narażonych na nieprzyjemności ze strony personelu medycznego powodu odmowy zaszczepienia dziecka. 13% rodziców szukało wsparcia u innych rodziców w podtrzymaniu tej decyzji.

Niemal połowa rodziców nie dzieliła się swoimi doświadczeniami z innymi rodzicami. Podobny odsetek rodziców polecałby innym nieszczepienie dziecka w okresie noworodkowym. Zaledwie 7% rodziców uchylających się od szczepień poleciłoby jednak zaszczepienie dziecka w oddziale noworodkowym (tab. V).

**Table III:** Premises of parents’ decision not to vaccinate a child in a neonatal unit (multiple answer option). Tabela III. Przesłanki decyzji rodziców o nieszczepieniu dziecka w oddziale noworodkowym (możliwość wielokrotnej odpowiedzi).

Treść *Contents*	Liczba odpowiedzi (%) *Number opf responses (%)*
Odroczenie szczepienia n=96	
*Postponement of vaccination n=96*	
Bezpieczniej dla dziecka	52 (54)
*Safer for the child*	
Lepsza odpowiedź immunologiczna	24 (25)
*Better immune response*	
Ryzyko zachorowanie nieduże	14 (15)
*The risk of getting the disease is small*	
Zostaliśmy przekonani, aby zaszczepić później	20 (21)
*We were convinced to vaccinate later*	

Rezygnacja (trwała odmowa) n=24	
*Resignation (permanent refusal) n=24*	
Brak informacji o korzyściach szczepień	7 (29)
*No information about the benefits of vaccination*	
Obawa o niekorzystne działanie szczepionki	12 (50)
*Concern about adverse effects of the vaccine*	
Brak wiary w skuteczność szczepień	3 (12,5)
*Mistrust of the effectiveness of vaccination*	
Obawa o interes komercyjny	5 (21)
*Suspicion of commercial interest*	
Niekorzystne doświadczenia ze szczepieniem w rodzinie	9 (37,5)
*Unfavorable experience with vaccination in the family*	

**Table IV:** The realization of vaccinations at 6 months of age in the group not vaccinated in the neonatal period. Tabela IV. Realizacja szczepień ochronnych w 6. miesiącu życia dziecka w grupie nieszczepionych w okresie noworodkowym.

Rodzaj decyzji *Type of decision*	Liczba odpowiedzi *Number of responses*
Odroczenie szczepienia	96 (80%)
*Postponement of vaccination*	
Odmowa szczepienia	24 (20%)
*Refusal of vaccination*	

**Table V:** Attitudes of parents for immunization on the day of the interview (multiple choice option). Tabela V. Postawy rodziców wobec szczepień w dniu rozmowy (możliwość wielokrotnego wyboru).

Treść	Liczba odpowiedzi (%)
*Content*	*Number of responses (%)*
Postawy wobec uprzednio podjętej własnej decyzji	
*Attitudes towards previously made own decision*	
zadowoleni	112 (93)
*glad*	
bez znaczenia	4 (3)
*irrelevant*	
żałujemy	0 (0)
*we regret*	
narażenie na nieprzyjemności	8 (7)
*exposure to unpleasantness*	
grupa wsparcia w innych rodzicach	16 (13)
*support group in other parents*	

Postawy wobec innych rodziców	
*Attitudes towards other parents*	
polecamy nieszczepienie	36 (30)
*we recommend non-vaccination*	
polecamy odraczanie	20 (17)
*we recommend postponement*	
polecamy zaszczepienie	8 (7)
*we recommend vaccination*	
bez komentarza, nie udzielamy rad	56 (47)
*without comment, we do not give advice*	

## Dyskusja

Uchylanie się od szczepień ochronnych jest narastającym zjawiskiem w oddziałach noworodkowych w Polsce i na świecie. Na przestrzeni zaledwie trzech lat, w naszej Klinice liczba rodziców nie szczepiących swoich dzieci uległa podwojeniu i osiągnęła wartość 2,83%. W skali Polski zjawisko odmowy zaszczepienia dziecka w oddziale noworodkowym dotyczyło w roku 2017 około 3000 noworodków, co stanowi ok. 0,7% wszystkich nowo urodzonych [8].

W naszym badaniu uchylającymi się od szczepień ochronnych byli w większości rodzice wykształceni, rodzicielsko dojrzali, posiadający więcej niż jedno dziecko. W tej grupie ponad połowa rodziców posiadała doświadczenie w szczepieniu poprzednich dzieci. Byli to rodzice aktywni zawodowo. Zestawienie tych cech mogłoby sugerować, że rodzice uchylający się od szczepień ochronnych zdobyli wiedzę na temat bezpieczeństwa szczepień ochronnych i towarzyszących im zdarzeń niepożądanych. Były to osoby o zdecydowanych poglądach i ugruntowanych przekonaniach. Dane te są zgodne z obserwacjami innych autorów. Według Opel i Gust około 5% rodziców odmawia kategorycznie zaszczepienia dziecka, zaś 30% należy do grupy wahających się [9, 10].

W Polsce, tak jak w innych krajach o wysokim dochodzie i o ustabilizowanym programie szczepień często twierdzi się, że szczepionki są „ofiarami własnego sukcesu”. Spadek liczby chorób zakaźnych przeciwko którym wykonywane są szczepienia ochronne powoduje, że rodzice nie mają z nimi bezpośredniego doświadczenia [11, 12]. Dlatego obawa przed ryzykiem związanym ze szczepionką może być bardziej widoczna niż obawa przed odpowiadającymi im chorobami. Argument ten nie tłumaczy jednakże spadku akceptacji niektórych lub wszystkich szczepionek w krajach o niskim i średnim dochodzie, gdzie schorzenia te nadal stanowią bardziej bezpośrednie zagrożenie dla zdrowia [13, 14, 15, 16].

Badani przez nas rodzice w dużym stopniu wiedzę na temat szczepienia ochronnego, a zwłaszcza nieszczepienia dziecka czerpią z internetu. Swobodny dostęp do niekontrolowanych treści o nieweryfikowanej wiarygodności i rzetelności naukowej sprzyja trendom antyszczepionkowym. Dostęp do stron antyszczepionkowych obniża motywację do realizacji programu szczepień ochronnych [17, 18, 19].

Niewielu rodziców oparło decyzję o nieszczepieniu na analizie literatury medycznej, od znajomych czy rodziny. Odroczenie szczepienia ochronnego wynikało z obaw o nieznany status immunologiczny dziecka w pierwszych dniach po urodzeniu i niemożliwą do przewidzenia reakcję na szczepienie. Jeszcze częściej obawiano się niedostatecznej odpowiedzi immunologicznej lub nadmiernej reakcji na podanie szczepionki. Część rodziców nie bardzo wierzyła w informację o realnym zagrożeniu zachorowaniem dziecka. Co piąty rodzic (20% grupy) po prostu biernie przyjął opinię o korzyściach odroczenia szczepienia.

Bardziej zdecydowani w poglądach okazali się rodzice trwale odmawiający szczepień ochronnych. Dominującym powodem odmowy było przekonanie o niekorzystnym działaniu szczepionki. Jednakże tylko u niektórych z nich przekonanie to miało oparcie w obserwacjach własnych lub w najbliższej rodzinie o występowaniu działań niepożądanych po szczepieniu. Wielu po prostu przyjęło taką wiedzę z obcych źródeł.

Aż jedna trzecia rodziców nie uzyskała rzetelnej wiedzy od pracowników ochrony zdrowia. To bardzo niepokojące, bo wskazuje na konieczność gruntownego szkolenia personelu medycznego i uaktualniania wiedzy opartej na rzetelnych badaniach naukowych i obserwacjach epidemiologicznych. Badanie ankietowe przeprowadzone wśród polskich studentów wykazało, że połowa z nich miała kontakt z ruchami antyszczepionkowymi. Zaledwie 50% studentów uczelni medycznych postrzega ruchy antyszczepionkowe jako niekorzystne. Wśród studentów uczelni niemedycznych odsetek ten jest jeszcze mniejszy i wynosi 33%. Ponadto, wiedza na temat szczepień, jak występowanie niepożądanych odczynów poszczepiennych, odporności zbiorowiskowej czy obecności tiomersalu w szczepionkach wymaga gruntownego uzupełnienia, zwłaszcza u studentów uczelni medycznych [20].

Jak wynika z komentarzy niektórych badanych rodziców, propagowanie szczepień ochronnych wynika nie tylko z przekonania o skuteczności szczepień w zapobieganiu chorobom. Wielu rodziców wyraża obawy, że podstawą tych działań jest prosty interes komercyjny producentów szczepionek, a nawet korzyści personelu medycznego.

Co ciekawe, odmowy szczepień ochronnych z reguły nie dotyczą noworodków przedwcześnie urodzonych. Wydaje się, że dłuższy kontakt rodziców z personelem medycznym oraz obserwacja długiego i często skomplikowanego procesu zdrowienia dziecka wytwarza pewien poziom ufności, sprzyjający akceptacji zaszczepienia dziecka. U długo hospitalizowanych wcześniaków także następne szczepienia (DTaP+ HIB+PCV) sąw wielu oddziałach neonatologicznych wykonywane w szpitalu. Jak wykazano w naszym poprzednim badaniu, przyjęcie takiej taktyki sprzyja zarówno realizacji szczepień ochronnych jak i terminowości ich wykonania [21]. Mimo to ponad połowa oddziałów neonatologicznych na Mazowszu nie wprowadziła szczepień ochronnych wcześniaków do procedur realizowanych podczas hospitalizacji [22]. Tymczasem, u noworodków przedwcześnie urodzonych z istniejącymi czynnikami podwyższającymi ryzyko wystąpienia zdarzeń niepożądanych, wykonanie szczepień w szpitalu jest szczególnie uzasadnione [23].

Zaobserwowano zjawisko konsekwentnego trwania rodziców w podjętej w oddziale noworodkowym decyzji odroczenia szczepienia lub zaniechania go w ogóle. Do 6. miesiąca życia dziecka 80% pierwotnie uchylających się rodziców zrealizowała program szczepień ochronnych. 20% rodziców nadal nie podejmuje takiej decyzji. Wszystko wskazuje na to, że będzie to decyzja trwała.

Znamienne jest, że nikt z rodziców nie żałował decyzji z okresu noworodkowego. Konsekwentne jej utrzymywanie było dla części rodziców źródłem nieprzyjemnych zachowań ze strony personelu medycznego. Postawa personelu medycznego aktywnie negująca uchylanie się od szczepienia nie jest finalnie korzystna dla powodzenia w realizacji PSO [7]. Należy jednak zauważyć dylemat pomiędzy wolnością decyzji w stosunku do uodparniania czynnego własnego dziecka a szkodą wynikającą z obniżenia odporności zbiorowiskowej. Dynamicznie zmieniająca się sytuacja społeczna związana z migracjami, rosnące prawa do wolności, popularność ruchów przeciwko szczepionkom i wynikająca z tego redukcja odporności zbiorowiskowej zmusza środowisko medyczne i rządy różnych krajów do refleksji nad nastawieniem do szczepień. Europejska Akademia Pediatrii, w oparciu o dowody naukowe, zdecydowanie potwierdza bezpieczeństwo szczepień ochronnych. Deklaruje współpracę z rządami i mediami w poszczególnych krajach w zakresie poprawy wszczepialności dzieci [24].

Wielu rodziców znajduje oparcie w innych rodzicach, z którymi utrzymują kontakt. Aż jedna trzecia rodziców włącza się w czynną aktywność antyszczepionkową, zachęcając innych rodziców do uchylania się od szczepienia dziecka. Chociaż liczebność ruchów antyszczepionkowych nie jest duża, to grupa ta wywiera duży negatywny wpływ na opinię rodziców, a nawet osób w rządach państw co do bezpieczeństwa szczepień ochronnych [25, 26]. Jednocześnie, ponad połowa nieszczepiących w okresie noworodkowym, zachowuje wstrzemięźliwość w dzieleniu się swoimi opiniami z innymi rodzicami.

Istnieją postulaty, aby odmowa szczepienia była równoznaczna z zobowiązaniem rodziców do pokrycia kosztów leczenia w razie wystąpienia infekcji. Przeciwnicy szczepień, respektując swoje prawo do odmowy szczepienia, świadomie braliby odpowiedzialność nie tylko za zdrowie swojego dziecka, ale też skutki finansowe w przypadku zachorowania [27].

Projekt został zrealizowany w szpitalu położonym w aglomeracji wielkomiejskiej, stąd grupa uczestników badania może nie być reprezentatywna dla przeciętnej populacji. Trzyletni okres badania to względnie krótki czas dla wykazania trwałości trendu narastania odmowy szczepień ochronnych. Ponadto, okres obejmujący pierwsze 6 miesięcy życia każdego pacjenta co prawda umożliwił obserwację wdrożenia programu szczepień, ale nie było możliwe określenie realizacji pełnego cyklu szczepienia podstawowego. Wydaje się potrzebna analiza obejmująca co najmniej pierwsze dwa lata życia dziecka, aby ocenić zarówno wdrożenie jak też terminowość realizacji programu szczepień ochronnych dziecka.

## Podsumowanie

Uchylanie się rodziców od szczepień ochronnych dziecka w okresie noworodkowym jest zjawiskiem narastającym i przez to niepokojącym. Decyzja rodziców została podjęta na podstawie przyjętych informacji z różnych, zazwyczaj nie naukowych źródeł. Rodzice wykazują dużą pewność co do słuszności podjętej decyzji. Jednocześnie brakuje prawidłowo udzielonej, profesjonalnej porady medycznej. Rodzice oczekują informacji o korzyściach szczepień ochronnych, jak też ryzyku zastosowania szczepionki. Równie ważne są informacje o schorzeniach objętych czynną profilaktyką.

Działania personelu medycznego negujące kompetencje rodzicielskie rodziców, stwarzanie sytuacji dla nich przykrych oraz jawne negowanie ich decyzji należy uznać za nieodpowiednie i przez to nieskuteczne. Z badań Tymińskiej i wsp. wynika, że nawet krótka 1-minutowa profesjonalna porada lekarska może być skuteczna w ułatwieniu rodzicom podjęcia decyzji o szczepieniu ochronnym dziecka [28]. Środki prawnego przymusu, o ile są konieczne, powinny być ograniczone do absolutnego minimum [29].

Rodzice wcześniaków posiadają świadomość choroby i zagrożenia życia dziecka, mają też możliwość obserwacji profesjonalizmu personelu medycznego podczas procesu leczenia. W efekcie rośnie wiarygodność i zaufanie, co skutkuje akceptacją wdrożenia programu szczepień ochronnych u dziecka. Ta obserwacja wydaje się kluczowa i powinna stanowić drogowskaz dla personelu medycznego do jednolitych i aktywnych działań promujących szczepienia ochronne w stosunku do całej populacji noworodków i ich rodziców.

## Wnioski

1. Istnieje konieczność powszechnego monitorowania liczebności, przyczyn i powodów uchylania się od szczepień ochronnych rodziców nowo urodzonych dzieci w Polsce.
2. Personel medyczny powinien znać i rozumieć postawy rodziców oraz być szkolony w rzetelnym i profesjonalnym udzielaniu informacji zarówno co do sposobu komunikacji z rodzicami jak i przekazywanych treści.
3. Dla zrównoważenia aktywności antyszczepionkowych, w propagowaniu szczepień ochronnych konieczna jest większa aktywność zarówno personelu medycznego jak i środowisk pozamedycznych, z wykorzystaniem różnorodnych dróg komunikacji.
